# Chitosan/β-glycerophosphate *in situ* gelling mucoadhesive systems for intravesical delivery of mitomycin-C

**DOI:** 10.1016/j.ijpx.2019.100007

**Published:** 2019-02-22

**Authors:** Oluwadamilola M. Kolawole, Wing Man Lau, Vitaliy V. Khutoryanskiy

**Affiliations:** a Reading School of Pharmacy, University of Reading, Whiteknights, PO Box 224, Reading, RG6 6AD Berkshire, United Kingdom; b School of Pharmacy, The Faculty of Medical Sciences, Newcastle University, Newcastle Upon Tyne NE1 7RU, United Kingdom

**Keywords:** Chitosan, β-Glycerophosphate, *In situ* gelling, Mucoadhesion, Intravesical drug delivery, Mitomycin-C

## Abstract

The development of mucoadhesive *in situ* gelling formulations for intravesical application may improve the therapeutic outcomes of bladder cancer patients. In this work, chitosan/β-glycerophosphate (CHIGP) thermosensitive formulations have been prepared using three different chitosan grades (62, 124 and 370 kDa). Their ability to form *in situ* gelling systems triggered by changes in temperature upon administration to urinary bladder were evaluated using vial inversion and rheological methods. Texture analysis was used to study their mucoadhesive properties as well as syringeability through the urethral catheter. The retention of CHIGP formulations, with fluorescein sodium as the model drug, was studied on porcine urinary bladder mucosa *ex vivo* using the flow-through technique and fluorescent microscopy. CHIGP formulations containing mitomycin-C were prepared and drug release was studied using *in vitro* dialysis method. It was established that the molecular weight of chitosan influenced the thermogelling, mucoadhesive and drug release behaviour of the *in situ* gelling delivery systems. Formulations prepared from chitosan with greatest molecular weight (370 kDa) were found to be the most promising for intravesical application due to their superior gelling properties and *in vitro* retention in the bladder.

## Graphical abstract

**Figure:**
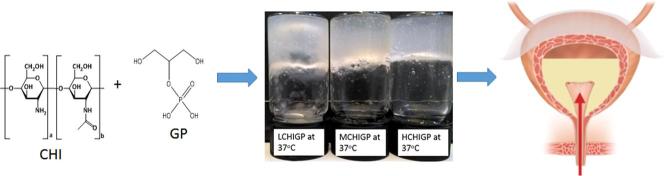

## 1 Introduction

Bladder cancer has been identified as a major clinical issue with prevalence and mortality rate escalating globally (Torre et al., 2015) and there is an increasing number of research in this area to improve drug delivery (Hu et al., 2018, Kolawole et al., 2017). Oral and other systemic routes of administration are not employed for bladder cancer treatment, especially at the early stages of the disease because therapeutic drug concentrations cannot be achieved in the bladder due to the hostile environment of the stomach, hepatic metabolism as well as drug transport to non-target organs (GuhaSarkar and Banerjee, 2010). Consequently, drugs are instilled via the catheter directly into the bladder, referred to as intravesical drug delivery, to increase local drug availability. Local route of administration has been explored further for bladder cancer treatment because it minimises systemic toxicity and facilitates targeted drug delivery to urothelial malignant tissues (GuhaSarkar and Banerjee, 2010, Kolawole et al., 2017).

Polymers exhibiting the ability to adhere to mucosal tissues in the bladder are typically referred as mucoadhesive. The mucoadhesive dosage forms are commonly prepared using polymers that interact with glycoprotein components of mucin through non-covalent bonding like hydrogen bonds, chain entanglements and electrostatic interactions (Khutoryanskiy, 2011, Davidovich-Pinhas and Bianco-Peled, 2014, Khutoryanskiy, 2014). They are particularly desirable for drug delivery to the bladder because they may be able to overcome some inherent limitations of intravesical administration such as substantial drug dilution and wash-out during urine formation and elimination.

Chitosan, (1,4)-2-amino-2-deoxy-β-D-glucan, is a natural polymer generated by the partial deacetylation of chitin under basic or enzymatic conditions (Ravi Kumar, 2000). It is commercially available as various grades depending on molecular weight and degree of deacetylation, with the highly deacetylated forms preferred because they can be readily functionalised for a variety of biomedical applications (Jayakumar et al., 2010). The continued interest in chitosan over the last two decades for drug delivery and tissue engineering is due to its biocompatible, biodegradable, mucoadhesive, and cell permeation properties (Bernkop-Schnürch and Dünnhaupt, 2012, Jayakumar et al., 2010). Tyagi et al reported that chitosan exhibited properties desirable for the formulation of intravesical dosage forms: effective and extended mucoadhesion as well as non-interference with bladder physiology (Tyagi et al., 2006).

Glycerophosphate is presented as its sodium salt which is hydrolysed in the body to inorganic phosphate and glycerol (U.S. National Library of Medicine, 2005). It has been approved by MHRA as a phosphate supplement in intravenous nutrition for adult patients marketed by Fresenius Kabi, which is a concentrate containing 21.6% sodium glycerophosphate for infusion (MHRA, 2016). The possibility of chitosan forming *in situ* gelling systems with physical cross-linking was first reported by Chenite et al. (2000), where chitosan solution (pH ∼ 6) formed a gel in the presence of β-glycerophosphate at about 37 °C. Chitosan/β-glycerophosphate mixtures were reported to be safe, biodegradable and thermosensitive with relatively easy drug loading that allow its release at the point of administration; plus their preparation does not require expensive equipment (Hastings et al., 2012, Kean and Thanou, 2010). They also display sustained gel stability for 90 days when stored at −80 °C (Peng et al., 2013).

Various researchers investigated one or two grades of chitosan or its derivatives with αβ- or β-glycerophosphate and reported improved efficacy and sustained drug release of various therapeutics (Abdel-Bar et al., 2014, Aliaghaie et al., 2012, Khodaverdi et al., 2012, Kim et al., 2010, Peng et al., 2013, Supper et al., 2013, Zheng et al., 2010, Zhou et al., 2015). Zhang and co-workers have mixed Bacillus Calmette Guérin (BCG) with ferric oxide (Fe_3_O_4_) nanoparticles and incorporated the nanoparticulate formulation into chitosan/β-glycerophosphate *in situ* gelling systems to generate composite system for intravesical bladder cancer treatment (Zhang et al., 2013). To the best of our knowledge, chitosan/β-glycerophosphate *in situ* gelling systems have not been explored for intravesical drug delivery. Moreover, the wash-out influence of artificial urine on the retention of *in situ* gelling drug carriers has not been studied. The drug-loaded *in situ* gelling formulations may form a mucoadhesive gel layer across a wide surface area of the bladder mucosa allowing for therapeutic concentrations of the drug to diffuse across urothelial cancerous tissues for extended period of time.

Mitomycin-C is the drug used for superficial/non-invasive bladder cancer therapy, administered preferably immediately or ≤24 h after transurethral resection of bladder tumor to reduce recurrence rate (Mostafid et al., 2006, NICE, 2015). Mitomycin-C is generally administered at a concentration of 1–2 mg/mL for superficial bladder cancer treatment (Kirin, 1992). According to a randomised controlled trial, 1 mg/mL mitomycin-C formulation (20–40 mL) resulted in recurrence reduction of 23.5% in bladder cancer patients (Au et al., 2001). So, mitomycin-C at 1 mg/mL was selected for the current study.

Bilensoy and co-workers explored cationic chitosan- and poly-l-lysine-coated poly-Ɛ-caprolactone (PCL) nanoparticles for improved intravesical delivery of mitomycin-C (Bilensoy et al., 2009, Erdoğar et al., 2014, Erdoğar et al., 2012). They reported that chitosan-coated PCL nanoparticles used to treat bladder tumour in rats *in vivo* displayed superior antitumour efficacy (evident with more rats alive up to 83 days) relative to other groups treated with poly-l-lysine-coated PCL nanoparticles and uncoated chitosan nanoparticles (Erdoğar et al., 2014). Their findings suggested that chitosan coated drug carriers may be efficient for improved drug localisation and accumulation in bladder tissues.

Despite the potential of *in situ* gelling systems to facilitate controlled drug release (Packhaeuser et al., 2004), they have not been explored as delivery systems for mitomycin-C in enhancing the therapeutic outcome of bladder cancer. This current work sought to explore chitosan-based *in situ* gelling systems to improve the residence time of mitomycin-C in the bladder. We present the first report on the formulation of chitosan/β-glycerophosphate gels using three grades of chitosan for intravesical drug delivery, with chitosan molecular weight modulating gelation, mucoadhesive and drug release profile of the CHIGP formulations.

## 2 Materials and methods

### 2.1 Materials

Low (LCHI), medium (MCHI) and high molecular weight (HCHI) grades of chitosan, β-glycerophosphate (β-GP), FITC-dextran (3–5 kDa), dextran (5 kDa), fluorescein sodium, trifluoroacetic acid, urea, uric acid, magnesium sulphate heptahydrate, sodium hydrogen phosphate, creatinine, sodium bicarbonate, sodium sulphate, disodium oxalate and trisodium citrate acid were purchased from Sigma-Aldrich (UK); disodium phosphate, sodium chloride, potassium chloride, ammonium chloride, and calcium chloride dihydrate, mitomycin-C, HPLC grade methanol, acetonitrile and water and other chemical reagents were from Fischer Scientific (UK) and used as received without further purification. Dialysis membranes with molecular weight cut off 12–14 kDa were supplied by Medicell International (UK). Freshly excised porcine urinary bladders were provided by PC Turner Abattoir (Farnborough, Hampshire, UK).

### 2.2 Characterisation of chitosan

The molecular weights of the three grades of chitosan were determined by gel permeation chromatography using 0.1 M sodium nitrate as a solvent system (pH 2.1) with a flow rate of 1 mL/min at 25 °C. Approximately 20 mg of LCHI, MCHI and HCHI were dissolved in 3 mL deuterium oxide (D_2_O) acidified with 30 µL trifluoroacetic acid for 12 h at room temperature and the ^1^H NMR spectra were recorded using 400 MHz ULTRASHIELD PLUS™ B-ACS 60 spectrometer (Bruker, UK). The degrees of acetylation of chitosan samples were evaluated based on the integration pattern of the N-acetyl protons (*δ* = 1.6 ppm) relative to the other protons (*δ* = 3.0–3.6 ppm). An exemplar ^1^H NMR spectrum is provided in Fig. S1 (Supplementary information).

The acetylation level was evaluated using the following equation (Sogias et al., 2008):

(1) $$\text{DA}\left(\%\right)=\left({\text{I}}_{\text{CH}3}/3\right)/\left({\text{I}}_{\text{H}2-\text{H}6}/6\right)\times 100\%$$

where the integral intensity of N-acetyl protons is denoted as I _CH3_ and I _H2-H6_ depicted the integral intensities of H-2, -3, -4, -5 and H-6 of the deacetylated glucosamine ring of chitosan.

### 2.3 Preparation of CHI and CHIGP formulations

1% w/v LCHI in 12% w/v β-GP (LCHIGP), 1% w/v MCHI in 12% w/v β-GP (MCHIGP) and 1% w/v HCHI in 12% w/v β-GP (HCHIGP) formulations were prepared according to a previously reported procedure with modification (Khodaverdi et al., 2012). Briefly, 1% w/v of chitosan solutions were prepared in 0.1 M acetic acid (buffered to pH 4 using 1 M potassium hydroxide) for 12 h at room temperature. The β-GP solutions (48% w/v, 2 mL) were added to chitosan solutions (6 mL) in a dropwise manner under ice-cold conditions, giving a final β-GP concentration of 12% w/v and chitosan to β-GP volume ratio of 3:1. Chitosan solutions were also prepared without β-GP for comparison.

### 2.4 Characterisation of CHIGP formulations

#### 2.4.1 pH determination

The pH of CHIGP solutions was measured using a pH meter (SevenEasy Mettler-Toledo). Data was expressed as mean ± S.D (n = 3).

#### 2.4.2 Zeta potential measurements

The zeta potential of the formulations was evaluated based on our previously reported method (Kolawole et al., 2018). Briefly, folded DTS-1070 capillary cells (Malvern, UK) were filled with 1% w/v chitosan solutions or CHIGP formulations (sol form) and their zeta-potential values were determined at 25 and 37 °C using Zetasizer Nano-ZS (Malvern Instruments, UK). The samples were studied after 1 in 20 dilution to 0.05% w/v chitosan: 0.6% w/v β-glycerophosphate using ultrapure water. The instrument was set to operate at an absorbance of 0.01 and refractive index of 1.59. Measurements were conducted in triplicates with 50 sub-runs per reading.

#### 2.4.3 Syringeability through the catheter

The ability of various formulations to pass through a catheter via a syringe was evaluated using TA-XT Plus Texture Analyser (Stable Micro Systems, UK) operated at a compression mode. Male SpeediCath® 28,414 CH 14/4.7 mm catheter (Coloplast A/S, Denmark) was used in this study. The experiment was carried out using a previously reported method with slight modification (Jones et al., 2000). Briefly, the samples were loaded into 2 mL plastic syringes connected to a catheter. The syringe was vertically secured while the probe was lowered until it had an initial contact with the syringe plunger. Then, the probe was lowered at a constant speed of 2 mm/s for 25 mm (Fig. S2, Supplementary information). The work done to expel the syringe contents (work of compression) at 25 °C was assessed as a function of the area under the force-distance curve recorded during the plunger compression (n = 3). Sodium chloride (0.9% w/v), which is typically used in the urology clinic to prepare mitomycin-C solutions for intravesical administration, served as the control.

#### 2.4.4 Gelation studies using vial inversion method

The gelation time of the formulations was evaluated at 37 °C using a modified version of a vial inversion method reported earlier (Khodaverdi et al., 2012). Briefly, 3 mL LCHIGP, MCHIGP, HCHIGP samples were incubated in glass vials in a temperature-controlled water bath (Grant Instruments, Ltd, Cambridge) at 37 °C. The vials were inverted at predetermined time intervals to evaluate the flow of the samples by visual examination. The gelation time was identified as the point where the formulations stopped flowing.

#### 2.4.5 Rheology

The viscoelastic properties of the formulations were evaluated using an AR-2000ex rheometer (TA Instruments, UK) operated at the oscillatory mode using 40 mm parallel plate and a trim gap of 0.4 mm. The samples were steadily deposited onto the lower plate of the rheometer and the chosen trim gap was applied to reduce sample shearing, with the solvent trap in place during sample analysis to prevent sample loss. In order to determine the linear viscoelastic region of the samples at 25 °C, a “strain sweep” was carried out, where the magnitude of strain applied on the samples was steadily increased from 0.05 to 10%, at a constant frequency of 1 Hz. The strain, where the storage/elastic modulus (G′) and loss/viscous modulus (G″) was unchanged and independent of the prevalent frequency, was chosen for the frequency sweep studies.

##### 2.4.5.1 Gel strength determination: Frequency sweep analysis

Based on the strain sweep analysis, 1% strain was chosen for the “frequency sweep” conducted at 25 °C with samples scanned from frequency of 0.01–10 Hz to confirm optimal frequency for the rheological experiment. In order to evaluate the gel strength of the formulations, a “frequency sweep” was carried out at 37 °C over the frequency range of 0.01–10 Hz, applying 1% strain. The gel strength of the samples was evaluated based on the ratio of their storage modulus (G′) to loss modulus (G″) at a frequency of 0.1 Hz. The higher the G′/G″ value, the stronger the gel and vice versa (Ström et al., 2014).

##### 2.4.5.2 Gelation temperature determination: Temperature ramp test

The gelation temperature of the samples was evaluated using a temperature ramp test with samples heated from 20 to 50 °C at 1 °C/min, frequency and strain of 1 Hz and 1%, respectively. The sol-gel transition temperature was the temperature, where a rapid increase in the magnitude of G′ relative to G″ as the samples are subjected to increasing temperature via the rheometer plate. The tangent of the loss modulus to storage modulus (tanδ) was also evaluated over the studied temperature range. LCHIGP, MCHIGP and HCHIGP samples were also evaluated for their storage modulus values at 37 °C during temperature ramp test as this rheological parameter depicted their elastic properties at physiological temperature which impacts their gelation potential.

##### 2.4.5.3 Gelation time determination: Time sweep analysis

The gelation time was evaluated by carrying out a time sweep experiment, with samples maintained at 37 °C for 30 min, applying a respective strain and frequency of 1% and 1 Hz. The gelation time is identified as the time, where there is a sharp increase in the G′ value relative to that of G″, when samples are maintained at 37 °C for predetermined length of time. The tangent of the ratio of the loss modulus to storage modulus (tanδ) over 30 min was also investigated. As some samples displayed similar gelation time during time sweep analysis but different gelation times recorded during the vial inversion method, the G′ values of the CHIGP systems (which correlates with their elastic properties) after 30 min of maintaining them at 37 °C were also evaluated.

### 2.5 Retention on porcine bladder: Urine wash-out experiment

The artificial urine used for *ex vivo* porcine retention and drug release studies was prepared according to a previously reported method (Chutipongtanate and Thongboonkerd, 2010). Briefly, the following compounds were dissolved in 2 L ultrapure water (18.2 MΩ): urea (24.27 g), uric acid (0.34 g), magnesium sulphate heptahydrate (1.00 g), sodium hydrogen phosphate (1.00 g), disodium phosphate (0.11 g), creatinine (0.90 g), sodium bicarbonate (0.34 g), sodium sulphate (2.58 g), disodium oxalate (0.03 g), trisodium citrate (2.97 g), sodium chloride (6.34 g), potassium chloride (4.50 g), ammonium chloride (1.61 g), and calcium chloride dihydrate (0.89 g). The resultant artificial urine had a final pH of 6.2 ± 0.2.

The mucosal retention of fluorescein sodium on porcine urinary bladder mucosa, in the presence of chitosan and CHIGP samples was investigated using fluorescent MZ10F microscope (Leica Microsystems, UK) coupled with an “ET GFP” filter and a Zeiss Imager with exposure time of 70 ms (A1/AxioCam MRm, 1296 × 966 pixels; 0.8 × magnification), according to a method earlier developed by our group (Mun et al., 2016) with slight modification. To ensure that the mucus layer was preserved on the bladder tissue, the study was carried out using freshly excised porcine urinary bladders stored on ice during transport from the abattoir to the laboratory, refrigerated (4 °C) and used within 24 h. Contact with the mucosal side of the bladder tissue was avoided during excision of the required bladder sections (about 1.5 × 2.5 cm) and rinsed with artificial urine solution (∼3 mL) prior to tissue imaging. The bladder tissue was placed on a 75 mm × 25 mm glass slide and maintained in an incubator at 37 °C during urine wash-out (Fig. S3, Supplementary information). Microscopic images were recorded on each tissue sections before and after applying ∼50 µL of sample as well as after each of the five washing cycles with 10 mL artificial urine/cycle at 2 mL/min. Image J software (Java 8, National Institute of Health, USA) was employed to evaluate the microscopic images, generating mean fluorescence values as a function of urine volume used for the wash-out. The normalised fluorescence intensity during each urine wash-out cycle is obtained by subtracting the background fluorescence intensity from the raw fluorescence intensity at the wash-out cycle of interest. The value “1” was used to depict the fluorescence intensity from the tissue before artificial urine wash-out. The WO_50_ values were determined using the polynomial fit of the wash-out graphs (Fig. S4, Supplementary information), which represents the volume of artificial urine needed to wash out 50% of the formulations.

### 2.6 Mucoadhesive properties of the formulations

The TA-XT Plus Texture Analyser (Stable Micro Systems Ltd, UK) coupled with a 5 kg load cell was used as an additional technique to study the mucoadhesive properties of the formulations. Chitosan solution (0.4% w/v) served as the positive control, while the negative control was dextran solution (0.4% w/v). Porcine bladder tissues were secured at the base of a cylindrical container. The bottom of the cylindrical container had a circular cut-out region (20 mm in diameter) exposing the mucosal surface of the bladder tissue. This container was screwed onto the probe of the texture analyser through a hole drilled on the lid of the container. Another bladder tissue was placed on a petri dish and coupled onto the lower platform of the texture analyser, exposing bladder mucosal surface (20 mm in diameter) as shown in Fig. S5. The tests were performed using an earlier reported equipment settings (Caló et al., 2016) with slight modification: pre-speed test 1.0 mm/s; test speed 0.1 mm/s; post-test speed 0.1 mm/s; applied force 0.05 N; contact time 120 s; trigger type auto; trigger force 0.1 N; and return distance of 10.0 mm. Bladder tissues were incubated at 37 °C for 5 min prior to the study and the samples (0.4 mL) were applied onto the exposed area of the bladder tissue secured onto the lower platform of the texture analyser. The probe was then lowered such that the blank tissue comes in contact with the formulation applied onto the tissue secured on the lower platform for 2 min after which the parameters of interest (detachment force and total work of adhesion) were determined as shown in Fig. S6 (Supplementary information). The Texture Analyser software (T.A. Exponent, Stable Micro Systems, UK) was used to record the force versus distance curves. The maximum force needed to detach tissue from formulation indicated the adhesive strength of the samples, while the total work of adhesion was evaluated from the area under the force versus distance curve (Boateng et al., 2013, Caló et al., 2016).

### 2.7 Mitomycin-C *in vitro* release experiment

#### 2.7.1 Preparation of mitomycin-C loaded CHIGP formulations

Mitomycin-C-loaded CHIGP formulations were prepared by dissolving 2 mg mitomycin-C in 1 %w/v chitosan solutions (1.5 mL) and vortexed for one minute before β-GP solution (48% w/v) was added dropwise under ice-cold conditions and stirred for a further 30 min, producing a final β-glycerophosphate concentration of 12% w/v (giving MMC/LCHIGP, MMC/MCHIGP, MMC/HCHIGP). Mitomycin-C-loaded LCHI, MCHI and HCHI samples were prepared without addition of β-GP. This method was used because earlier studies reported that drug containing formulations exhibit superior sustained release profile relative to formulations, where drug was incorporated into the CHIGP mixture (Abdel-Bar et al., 2014, Zhou et al., 2008).

#### 2.7.2 Mitomycin-C *in vitro* release efficiency

*In vitro* drug release studies were carried out using a modified method used by Senyiğit et al. (2015). Briefly, 2 mL mitomycin-C loaded CHI and CHIGP solutions were placed in dialysis membrane bags (12–14 kDa MWCO) and allowed to gel in a water bath at 37 °C for 1 h. The dialysis bags with the gelled samples were placed in a stoppered 100 mL glass bottle containing 40 mL of artificial urine (pH 6.2 ± 0.2), maintained in a shaker water bath at 37 °C (60 rpm). At predetermined time intervals (0, 0.5, 1, 2, 4, 6, and 24 h), 1 mL of artificial urine was taken and replenished with same amount of fresh artificial urine. Drug content was analysed using previously reported HPLC-UV method with slight modification (Myers et al., 2017). The HPLC instrument was coupled with the quaternary pump and VWD UV detector (Agilent, Germany) operated at 365 nm. The aliquot samples (10 µL) were injected into the reverse phase C_18_ column, 150 mm × 4.6 mm, 5 µM (Dionex™, Thermo Scientific, UK) maintained at 25 °C. The mobile phase consisted of 83.5% of 25 mM sodium phosphate (pH 5.4) and 16.5% of methanol/acetonitrile (1:1), which was run in an isocratic mode at a flow rate of 1.5 mL/min, with run time of 15 min. Mitomycin-C eluted at ≈10 min, depicted on a typical chromatogram, Fig. S7 (Supplementary information). The standard curve of mitomycin-C was generated by analysing eight standard solutions of known concentrations.

### 2.8 Statistical analysis

All studies were carried out in triplicates, data expressed as mean ± SD and statistical differences were determined using *t*-test and One-Way ANOVA/post-hoc Bonferroni test with GraphPad Prism (version 5.04, USA) with p < 0.05 implying statistical significance.

## 3 Results and discussion

### 3.1 Characterisation of chitosan and chitosan/β-glycerophosphate formulations

According to the gel permeation chromatography data, the low (LCHI), medium (MCHI) and high (HCHI) molecular weight chitosan grades were 62, 124 and 370 kDa, with polydispersity indices (PDI) of 3.43, 3.54 and 6.98, respectively. The degrees of deacetylation were 82 ± 1%, 72 ± 2%, and 71 ± 2%, respectively, which are in good agreement with those used by previous researchers to formulate CHIGP *in situ* gelling systems (Supper et al., 2014, Zhou et al., 2015).

**Fig. 1:**
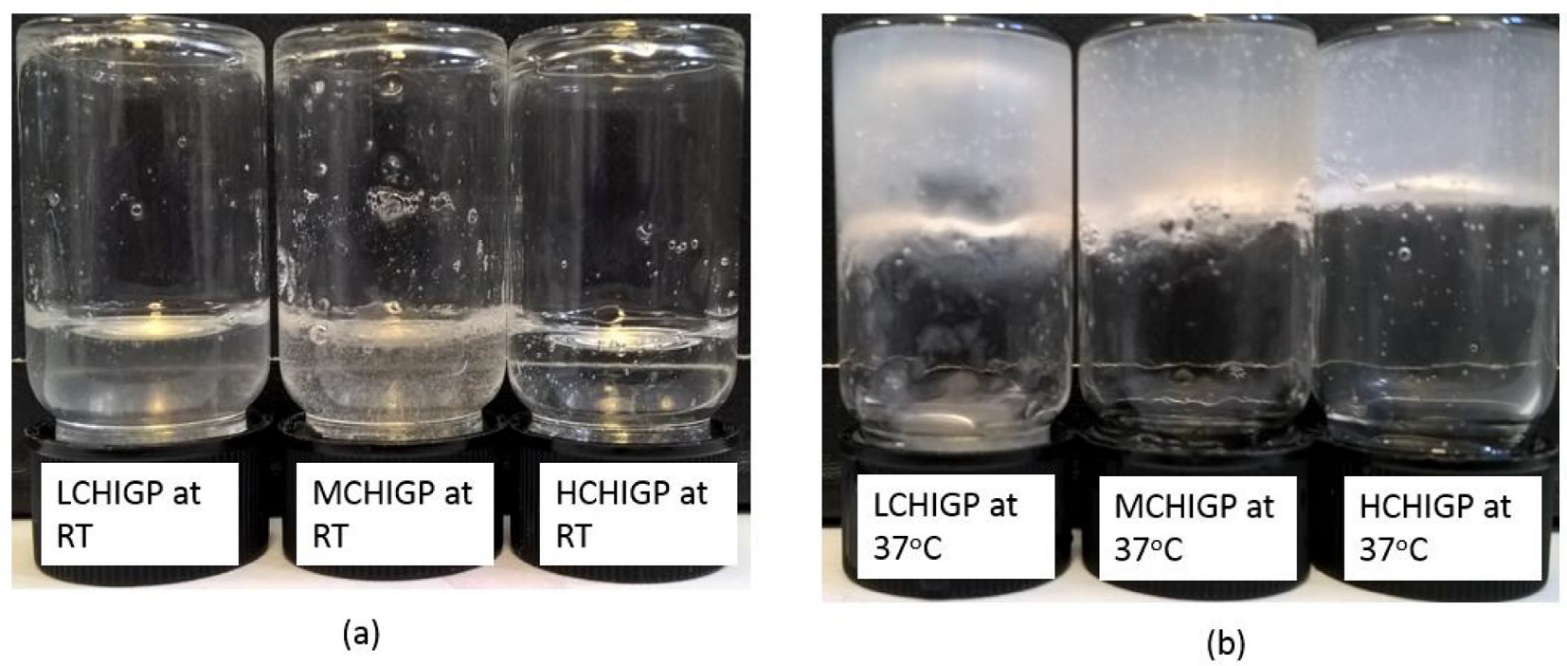
Exemplar images of chitosan/ß-glycerophosphate formulations at (a) room temperature and (b) 37 °C.

The focus of this study was to develop and characterise thermoresponsive and mucoadhesive formulations using chitosan/β-glycerophosphate mixtures. LCHIGP, MCHIGP and HCHIGP were formulated to contain 1% w/v chitosan, 12% w/v β-glycerophosphate and chitosan to β-glycerophosphate volume ratio of 3:1. All these formulations formed transparent solutions (pH 7.1–7.3) below 37 °C and turned into cloudy gels at 37 °C (Fig. 1). Chitosan/β-glycerophosphate mixtures are transparent below physiological temperature due to electrostatic attraction between negatively charged phosphate groups of β-glycerophosphate and positively charged ammonium groups of chitosan as well as hydrogen bonding involving chitosan functional groups (Chenite et al., 2001). Alcohol groups of glycerophosphate provide additional hydration due to hydrogen bonding with water molecules, thereby preventing gel formation below physiological temperature (Ruel-Gariépy et al., 2000). An increase in temperature up to 37 °C results in partial dissociation of hydrogen bonds with water molecules, leading to the formation of less hydrated gel.

All chitosan/β-glycerophosphate mixtures showed fast *in situ* gelation as detected using vial inversion method. MCHIGP and HCHIGP formulations formed physical gel within 7 ± 2 min and 5 ± 1 min, respectively, while LCHIGP mixture formed very weak gel at 15 ± 5 min that eventually collapsed upon further incubation at 37 °C (Fig. 1).

Despite the fact that LCHI had the greatest extent of deacetylation and a favourable pH of 7.4 ± 0.2 (Table 1), its gelation was not sustained. One of the reasons for this behaviour is the reduced degree of entanglements in LCHIGP, resulting in lower viscosity at increased temperature (Aliaghaie et al., 2012). This finding is in contrast with the study by Khodaverdi et al. (2012), who reported fast onset of gelation with highly deacetylated chitosan as a result of their assessment of medium weight chitosan. This finding proved that the molecular weight of chitosan played a more remarkable influence on their gelling properties than the degree of deacetylation of chitosan used in preparing the *in situ* gelling formulations.

**Table 1:** Rheological properties of CHI/ß-glycerophosphate samples.

Samples	pH	Frequency sweep, 37 °C	Temperature ramp (20–50 °C, 1 °C/min)			Time sweep for 30 min at 37 °C (Pa)			
		G′/G″ ratio at 0.1 Hz	Gelation temp (^o^C)	G′ at 37 °C (Pa)	tanδ at 37 °C	Gelation time (min)	G′ after 30 min at 37 °C (Pa)	tanδ after 30 min at 37 °C	
LCHIGP	7.4 ± 0.2	12.9 ± 1.8	30.4 ± 0.3	38.5 ± 3.5	0.04 ± 0.01	1.6 ± 0.3	30.2 ± 0.3	0.03 ± 0.01	
MCHIGP	7.5 ± 0.2	15.8 ± 0.1	29.8 ± 0.2	41.1 ± 2.2	0.03 ± 0.01	1.4 ± 0.3	59.7 ± 7.1	0.02 ± 0.03	
HCHIGP	7.3 ± 0.2	16.7 ± 0.2	29.6 ± 0.1	95.8 ± 5.5	0.03 ± 0.01	1.0 ± 0.1	138.0 ± 7.9	0.02 ± 0.02	

^*^Greater G′/G″ values at 0.1 Hz (frequency sweep at 37 °C) infer stronger gels; storage modulus (G′) values at 37 °C during temperature ramp test as well as G′ after 30 min during time sweep test depict elastic property and correlate with their ease of gelation. Loss factor or tanδ was calculated as tanG″/G′ ratio; when these values closest to zero this indicates greater ease of gelation (n = 3).

### 3.2 Rheological studies of gelation

This study was carried out to understand the structural and dynamic features of chitosan/ß-glycerophosphate formulations. During rheological analysis, samples were directly in contact with the heated rheometer plate at the temperature of interest, which simulated the physiological conditions of the bladder. A strain of 1% and frequency of 1 Hz were selected for the current rheological study so that the storage modulus (G′) and loss modulus (G″) of the samples were independent of the applied strain.

Frequency dependent rheological profiles of 1% w/v pure chitosan solutions are characteristic of viscous liquids, where G′ is lower than G″ at a particular frequency. This behaviour was evident with all studied chitosan samples (LCHI, MCHI and HCHI) as G″ remained greater than G′ at 25 and 37 °C during frequency sweep analysis, inferring the absence of gelation (data not shown). This data is in good agreement with the report by Supper et al (2013), where G′ was lower than G″ during frequency sweep studies carried out with 1.5% w/v chitosan solutions at 20, 30 and 40 °C.

**Fig. 2:**
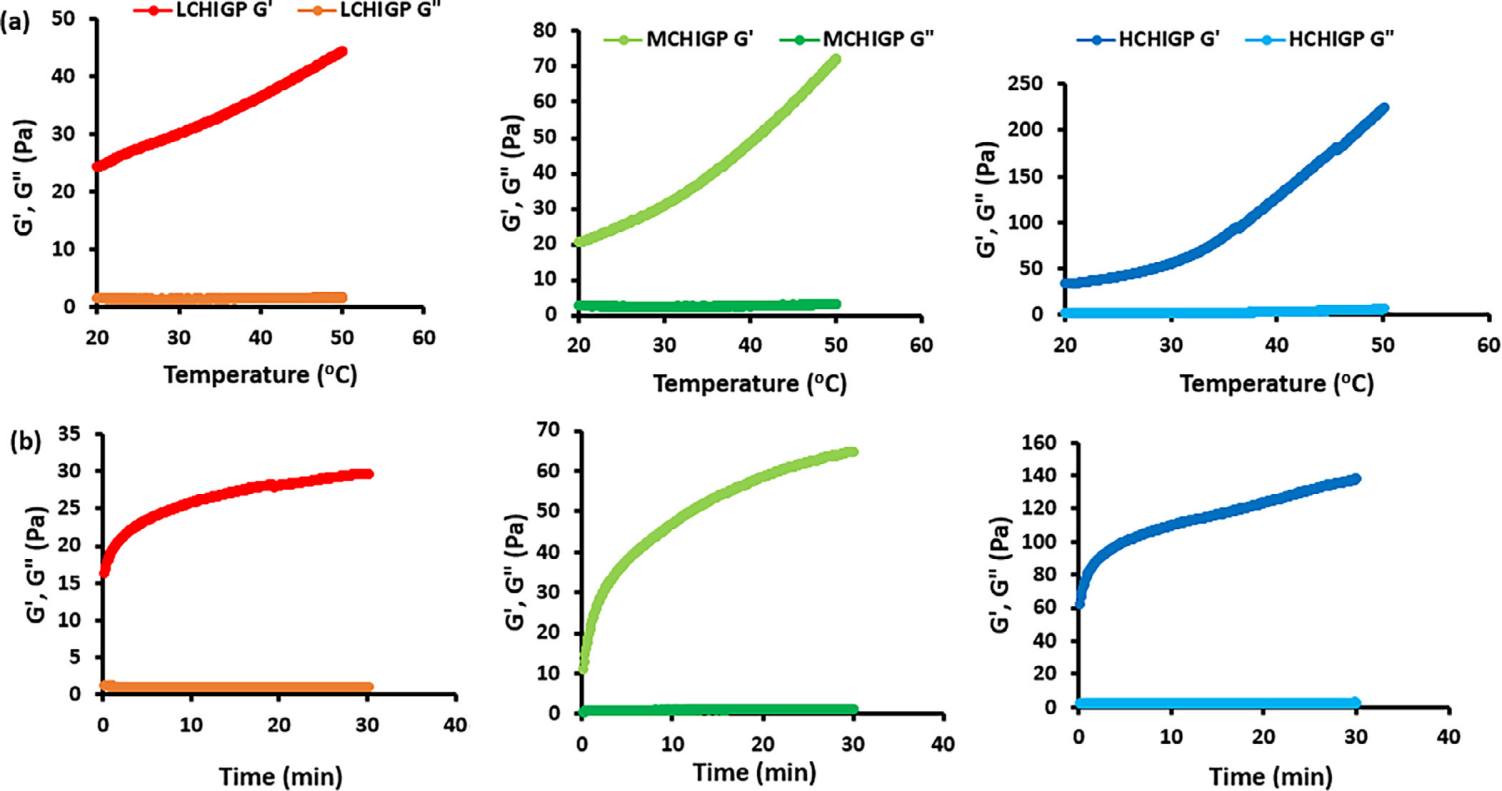
Exemplar rheological profiles of LCHIGP (red), MCHIGP (green) and HCHIGP (blue) showing (a) the temperature-dependent changes in viscoelastic properties at ramp rate 1 °C/min; (b) time-dependent viscoelastic changes of samples maintained at 37 °C for 30 min. (For interpretation of the references to colour in this figure legend, the reader is referred to the web version of this article.)

Gels typically display solid-like mechanical profiles, where the storage modulus (G′) is greater than the loss modulus (G″) throughout the evaluated frequency ranges. LCHIGP, MCHIGP and HCHIGP displayed gel-like behaviour at the onset of all rheological analysis (Fig. 2). This behaviour is desirable as it supports the rapid gelation of the samples at physiological temperature. Moreover, drug incorporation into the CHIGP formulations as well as urine presence in the bladder will potentially increase their gelation temperature and time.

With frequency sweep analysis at 37 °C, the greater the similarity in the values of G′ and G″, the weaker the gel. On the other hand, stronger gels exhibit rheological profiles, where the elastic modulus (G′) is greater than the viscous modulus (G″) (Ström et al., 2014). HCHI based formulations displayed superior gel strength (in terms of their G′/G″ values at frequency of 0.1 Hz during frequency sweep at 37 °C) relative to MCHI and LCHI based samples (Table 1). There was statistically significant difference between the gel strength of LCHIGP and MCHIGP as well as between LCHIGP and HCHIGP (p < 0.05), but the gel strength of MCHIGP and HCHIGP was similar (p > 0.05). This finding indicated that both MCHIGP and HCHIGP formulations may be potentially less susceptible to rapid erosion by urine in the bladder.

The temperature ramp analysis was conducted from 20 to 50 °C because this range was sufficient to study the transition of the drug carrier from the sol (≤25 °C) to the gel state (25–37 °C). Formulations intended for intravesical delivery ideally should have a gelation temperature of 30–36 °C (Choi et al., 1998). This ensures that such drug carriers remain liquid at room temperature during injection through the catheter and only transform into a gel within the bladder. *In situ* gelling systems with gelation temperature above 37 °C are not suitable for intravesical administration as they could be readily washed out of the bladder during urine voiding since they will remain liquid at physiological temperature.

For predominantly viscous materials, G″ is initially lower than G′ during temperature ramp test but as temperature increases, G″ increases at a faster rate than G′ and the sol-gel transition temperature is attained at the point of G″ and G′ intersection (Lin, 2002). Alternatively, gelation temperature is identified as the temperature, where there was a greater growth rate of G′ (elastic property) relative to the G″ (viscous property) without samples necessarily displaying a cross-over of G′ and G″ (Kim et al., 2010). This method of evaluating gelation temperatures may be explored for CHIGP formulations with medium viscosity showing a rheological profile with storage modulus G′ greater than the loss modulus G″ at the onset of the “temperature ramp” test with no possibility of G′ and G″ intersection at any studied temperature (Fig. 2). Moreover, some researchers have acknowledged that G′/G″ cross-over point during temperature ramp test may not depict the actual gelation temperature of the material (Aliaghaie et al., 2012, Madbouly and Ougizawa, 2006).

Our current study revealed that the temperature ramp profiles of some formulations may imply that they have similar gelation temperatures, which may not necessarily correlate with their ease of gelation using a different method of evaluating their thermogelation such as vial inversion studies at 37 °C. The gelation temperatures of LCHIGP, MCHIGP and HCHIGP formulations determined by rheological studies were 30.4 ± 0.3 °C, 29.8 ± 0.2 °C, and 29.6 ± 0.1 °C, respectively (p > 0.05). LCHIGP, MCHIGP and HCHIGP displayed similar gelation temperature based on the temperature at which there was a rapid change in their tanδ values (Fig. S8, Supplementary Information). So, there was the need to define the elastic potential of our CHIGP formulations based on their G′ and tanδ values at physiological temperature of 37 °C (Table 1). HCHIGP displayed significant greater extent of elastic features (95.8 ± 5.5 Pa) relative to MCHIGP (41.1 ± 2.2 Pa) and LCHIGP (38.5 ± 3.5 Pa) (p < 0.05). Similarly, tanδ values of MCHIGP and HCHIGP were smaller than that of LCHIGP, inferring that chitosan molecular weight has substantial effect on the ease of gelation of CHIGP *in situ* gelling systems despite the fact that MCHIGP and HCHIGP displayed similar tanδ values during temperature ramp and time sweep test.

Based on rheological time sweep at 37 °C for 30 min (Fig. 2b), the gelation times of LCHIGP, MCHIGP and HCHIGP were found to be 1.6 ± 0.3, 1.4 ± 0.3 and 1.0 ± 0.1 min, respectively, with HCHIGP forming gel most readily. As there was no significant differences in their gelation time based on their sharp increase in G′ relative to G″ as well as their tanδ values at any particular time, G′ values of the gels as well as their tanδ values were determined after maintaining the samples at 37 °C for 30 min. HCHIGP displayed a 2.3-fold and 4.3-fold increase in elastic features, relative to MCHIGP and LCHIGP, respectively. This result indicated that chitosan molecular weight influenced the gelation potential and gel strength of the formulations with β-glycerophosphate. This finding is in good agreement with vial inversion data for MCHIGP and HCHIGP formulations. In contrast, the results observed for LCHIGP differs as it formed gel less readily and the gel reversed to its sol state upon prolonged incubation at 37 °C (Fig. 1), whereas temperature ramp and time sweep studies indicated that it displayed similar gelation temperature and time with that of MCHIGP and HCHIGP formulations. Nevertheless, gel strength evaluation confirmed that LCHIGP was the weakest gel. We conclude that the elastic modulus (G′) measured at 37 °C may be a useful parameter for evaluating the gelation potential of CHIGP formulations, in addition to the already established techniques used in rheology to determine gelation temperature and time: (1) G′/G″ intersection evaluated during temperature ramp and time sweep test; (2) temperature and time where there is a rapid increase in G′ relative to G″.

It should be noted that all these rheological experiments were performed with the samples that were not diluted with urine. Following the intravesical administration of these *in situ* gelling formulations a dilution with urine is expected. This could potentially affect the gelation time. The effect of dilution of these formulations with urine was evaluated in a later section, describing retention on the bladder mucosa.

### 3.3 Syringeability through the urethral catheter

Syringeability is a critical parameter for evaluating the efficiency of intravesical dosage forms. A formulation that could flow through the catheter readily to quickly reach the bladder is one of the desirable attributes for intravesical delivery. Since administration of intravesical formulations is usually carried out at an ambient temperature, the syringeability test of our samples was conducted using a texture analyser at 25 °C.

**Fig. 3:**
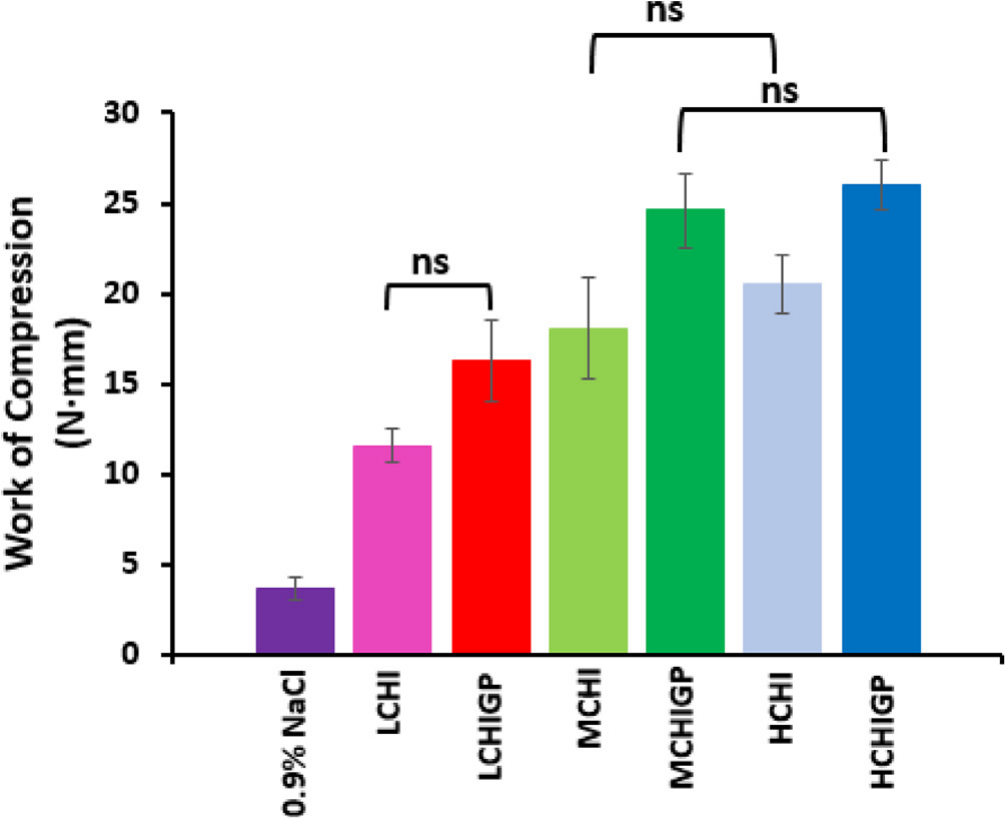
Syringeability of chitosan and chitosan/ß-glycerophosphate formulations evaluated as the work done to expel samples from 2 mL plastic syringes into the urethral catheter. Experiment was conducted at 25 °C using the Texture Analyser (Stable Micro Systems Ltd, UK), n = 3. Syringeability is inversely proportional to the work of compression; there was significant statistical difference in the work of compression values between all groups of samples (p < 0.05) except those designated with “ns”. 0.9% sodium chloride solution served as the control.

Fig. 3 shows the values of work required to release chitosan and CHIGP formulations from the syringe through a catheter, which is inversely proportional to syringeability of these formulations. The values for the work of compression of 0.9% NaCl, LCHI, MCHI, HCHI, LCHIGP, MCHIGP, HCHIGP were 3.75 ± 0.62 N·mm, 11.60 ± 0.94 N·mm, 18.07 ± 2.80 N·mm, 20.54 ± 1.63 N·mm, 16.29 ± 2.24 N·mm, 24.62 ± 2.05 N·mm, and 26.03 ± 1.38 N·mm, respectively. Sodium chloride (0.9% w/v), which is typically used to dissolve mitomycin-C for intravesical applications, displayed the lowest work of compression amongst all studied samples, implying that it was the most syringeable. There was statistically significant difference between the work of compression of 0.9% sodium chloride solution and all the other studied samples (p < 0.05). Chitosan molecular weight had a strong influence on the syringeability of the samples as LCHI with or without β-glycerophosphate were more syringeable than MCHI, MCHIGP, HCHI and HCHIGP (p < 0.05). However, blank and β-glycerophosphate containing MCHI and HCHI samples displayed similar work of compression (p > 0.05). This may be related to a gradual growth in chitosan molecular weight of 62 kDa, 124 kDa and 370 kDa for LCHI, MCHI and HCHI, respectively, as greater molecular weight of macromolecules results in higher solution viscosity and better resistance to flow. Also, the decreased syringeability may become less remarkable with further increase in chitosan molecular weight evident with MCHI and HCHI based samples. It should be noted that all values of the work of compression determined for the CHI and CHIGP systems (14–26 N·mm) were lower than the syringeability of chitosan and poloxamer gel based formulations reported in the study of Senyiğit et al (30–130 N·mm), indicating that our formulations are also syringeable.

### 3.4 Retention study on bladder tissue

The ability of polymeric dispersions to flow and adhere onto mucosal membranes is dependent on the surface energy between the drug carrier and mucosal surface. *In situ* gelling systems possess lower surface energy than that of the mucosal surfaces and readily spread over them, thereby maximising the contact area and optimising adhesion (Smart, 2005). Thus such systems are desirable to promote urothelial mucoadhesion and resistance to urine wash-out.

**Fig. 4:**
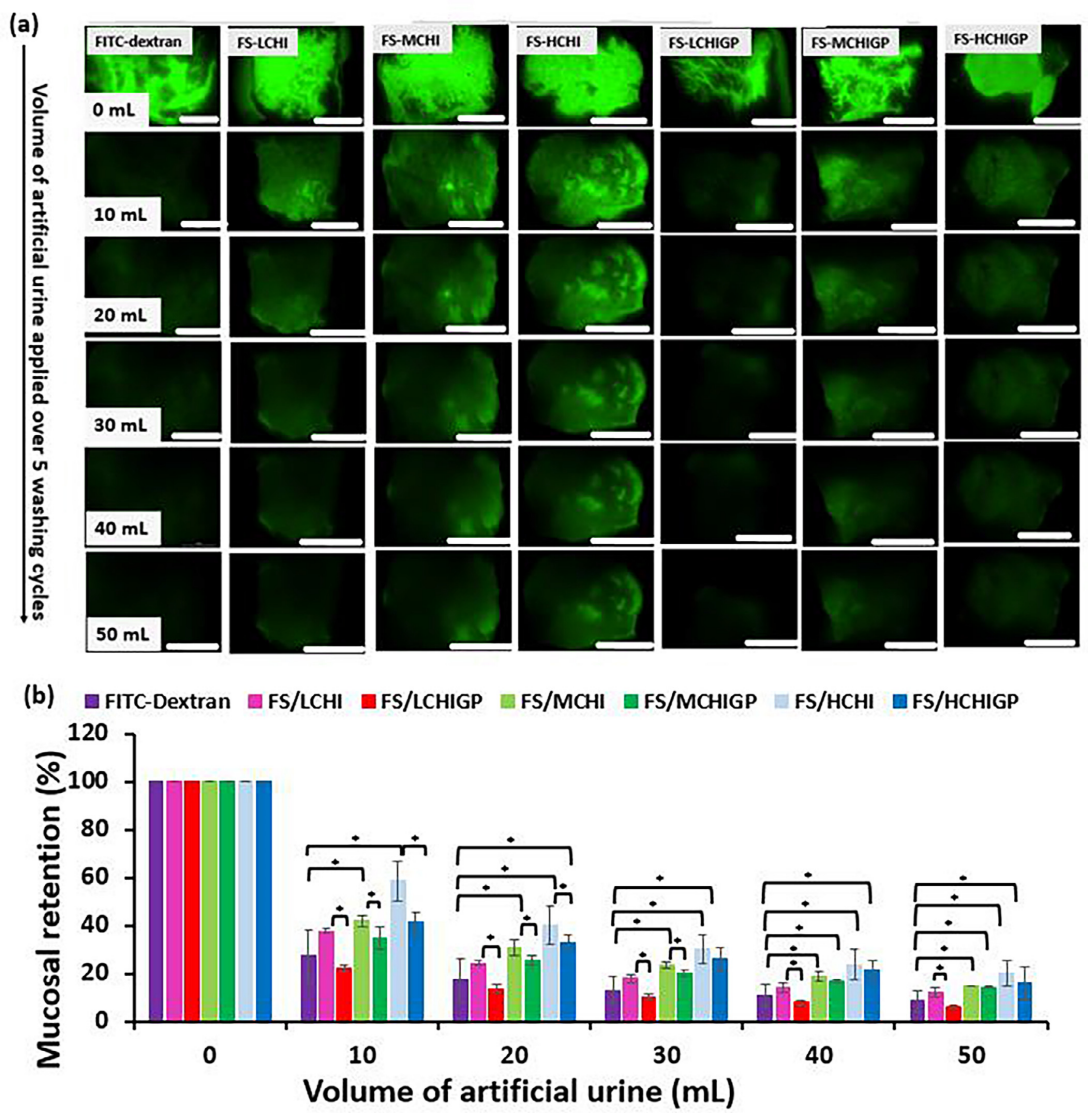
Retention study of fluorescein sodium (FS) formulations and FITC-dextran as a negative control. (a) Exemplar microphotographs showing wash-out from porcine urinary bladder tissue with artificial urine solution over 5 washing cycles, scale bar is 2 mm; (b). Mucosal retention on porcine urinary bladder tissue. Result presented as mean ± standard deviation, n = 3, * depicts statistically significant differences between samples (p < 0.05).

*Ex vivo* porcine urinary bladder retention studies were performed with fluorescein sodium as a model compound formulated using both chitosan solutions and their mixtures with ß-glycerophosphate. FITC-dextran (3–5 kDa) was also used in these experiments as a negative control due to well-known poor adhesiveness of this oligomeric polysaccharide to mucosal tissues (Štorha et al., 2013). WO_50_ is defined as the volume of biologically relevant fluid (simulated urine) required to wash out 50% of the fluorescent formulation from mucosal surface (Mun et al., 2016). Fig. 4 presents the results of these wash off experiments in the form of fluorescent images. The relative fluorescence intensity values were used to calculate the wash out_50_ values (WO_50_) of the formulations. It is clearly seen that FITC-dextran was removed from the surface of bladder mucosa with the first 10 mL of simulated urine, which is consistent with our previous reports (Kaldybekov et al., 2018, Kolawole et al., 2018, Mun et al., 2016). Formulations composed of chitosans and fluorescein sodium demonstrated excellent retention performance on the bladder surface with HCHI displaying superior resistance to urine wash out compared to its lower molecular weight analogues (p < 0.05). The formulations containing ß-glycerophosphate displayed reduced mucoadhesive properties relative to their respective chitosan solutions. For example, WO_50_ value for HCHI (14 mL) was significantly higher compared to its HCHIGP formulation (9 mL). This was a surprising result as one would expected the combination of excellent mucoadhesive properties of chitosan with formation of a gel *in situ* would provide a synergistic or enhanced retention effects on mucosa (Zahir-Jouzdani et al., 2018). However, this is not the case.

There was no significant difference between the fluorescence retention profiles of FITC-dextran and FS/LCHI, FS/LCHIGP, FS/MCHIGP and FS/HCHIGP after washing with 10 mL of artificial urine (p > 0.05), but the mucoadhesiveness of FS/MCHI and FS/HCHI was greater than that of FITC-dextran depicted by fluorescence intensity (Fig. 4a & b) (p < 0.05). With 20 mL artificial urine washing, the fluorescence retention of FS/HCHIGP was greater than that of FITC-dextran (p < 0.05). The significant difference between the mucoadhesiveness of FITC-dextran and MCHIGP was evident after 4 washing cycles with 40 mL artificial urine (p < 0.05). The mucoadhesive properties of FS/LCHI and FS/LCHIGP were similar to that of FITC-dextran after washing with 50 mL artificial urine (Fig. 4a & b).

**Fig. 5:**
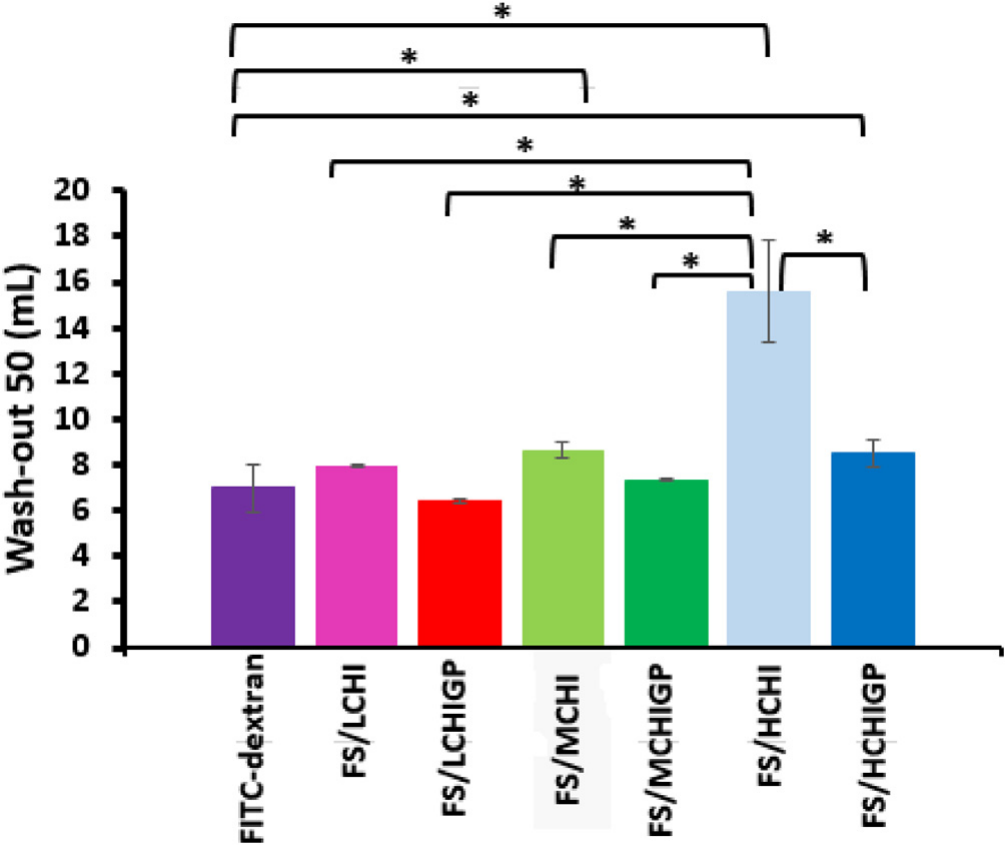
Artificial urine wash out-50 (WO_50_) values determined for different formulations. Results presented as mean ± standard deviation, n = 3, * depicts statistically significant differences between samples (p < 0.05).

Furthermore, FS/LCHI, FS/LCHIGP and FS/MCHIGP displayed similar WO_50_ values with FITC-dextran (p > 0.05). On the other hand, FS/MCHI, FS/HCHI and FS/HCHIGP were more mucoadhesive than FITC-dextran based on their WO_50_ values (Fig. 5). This finding demonstrated that high molecular weight chitosan is the most efficient grade to formulate CHIGP delivery systems for intravesical administration.

### 3.5 Mucoadhesive properties tested using tensile method

**Fig. 6:**
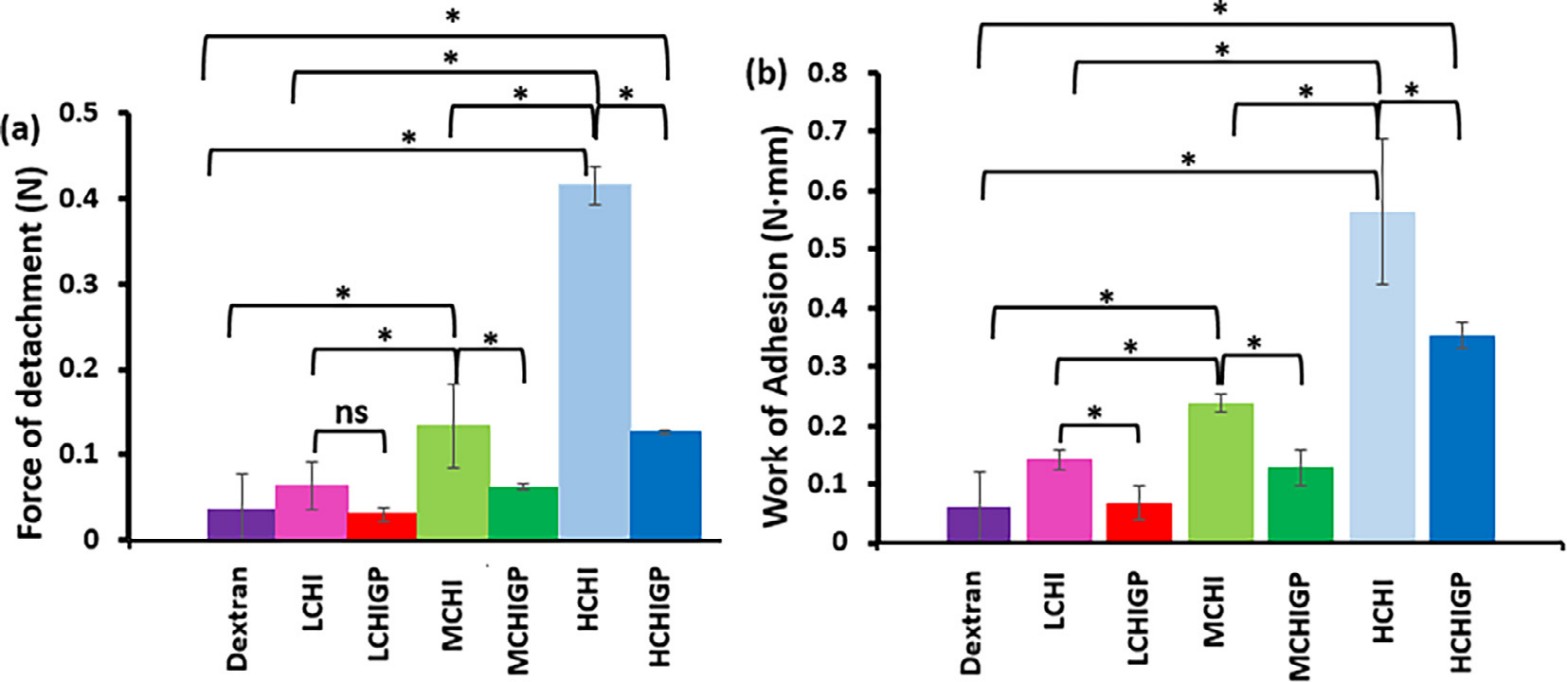
Adhesion of CHI (1% w/v) and CHIGP samples to porcine bladder mucosa using tensile method: (a) force of detachment values; (b) work of adhesion values. Results presented as mean ± standard deviation, n = 3, Asterisk (*) implies statistically significant difference between data sets (p < 0.05).

The tensile method was used to probe the mucoadhesive properties of the formulations further (Khutoryanskiy, 2011). In these experiments 0.4 mL of each liquid formulation was placed between two different bladder mucosal surfaces and, after holding these tissues in contact for 120 s, they were withdrawn from each other, recording the force versus distance profiles. The maximal force of detachment determined in these experiments indicated the force needed to surmount the adhesive bonds between the formulations and the bladder mucosa, while the area under the force-distance curves gave the total work of adhesion (Boateng et al., 2013, Caló et al., 2016). Typical detachment profile is presented in Fig. S6 (Supplementary information). As it was expected, dextran as the non-mucoadhesive control displayed the lowest force of detachment and total work of adhesion values (Fig. 6). Pure chitosan samples had a greater mucoadhesive performance compared to CHIGP formulations. For example, the values of maximal detachment force and total work of adhesion determined for HCHI (0.41 ± 0.02 N and 0.56 ± 0.13 N·mm, respectively) were significantly greater (p < 0.05) than the values for HCHIGP (0.13 ± 0.01 N and 0.35 ± 0.02 N·mm, respectively).

The detachment characteristics of all formulations correlate well with their resistance to urine wash-out: the use of both methods indicates greater mucoadhesiveness of chitosan samples compared to their mixtures with ß-glycerophosphate.

To get a further insight into the reasons why chitosan solutions alone show superior retention and mucoadhesive properties compared to the formulations of chitosan with ß-glycerophosphate, additional experiments were performed by determining zeta potential of all samples at 25 °C and 37 °C (Table 2). The chitosan formulations displayed a relatively high positive values of zeta potential (ZP) due to the cationic nature of chitosan associated with the presence of free amino groups. For the formulations with β-glycerophosphate, ZP values significantly decreased compared to CHI (p < 0.05). This trend was observed both at 25 °C and 37 °C. A significant reduction in ZP values is related to partial neutralisation of cationic chitosan macromolecules with anionic β-glycerophosphate, which is in good agreement with the literature data (Owczarz et al., 2018).

**Table 2:** Zeta potential values of chitosan solutions (CHI) and chitosan/ß-glycerophosphate mixtures (CHIGP) at 25 °C and 37 °C.

Chitosan Samples	Zeta potential, 25 °C (mV)		Zeta potential, 37 °C (mV)	
	CHI	CHIGP	CHI	CHIGP
Low	43.9 ± 2.7	1.5 ± 0.1	46.0 ± 1.6	1.1 ± 0.2
Medium	50.1 ± 7.8	1.9 ± 0.1	50.0 ± 2.7	1.2 ± 0.2
High	56.6 ± 2.3	2.3 ± 0.1	51.8 ± 0.3	1.6 ± 0.2

It is well established that excellent mucoadhesive properties of chitosan are related to the interactions of its macromolecules with negatively charged mucin. The nature of these interactions is predominantly electrostatic; however, the contribution of hydrogen bonding and hydrophobic effects cannot be completely disregarded (Sogias et al., 2008). A reduction in the positive values of zeta potential observed in the case of CHIGP formulations compared to chitosan alone may well be the main reason for their decreased interactions with bladder mucosal surfaces. Second factor that could possibly contribute to weakening of mucoadhesive performance is the physical cross-linking of chitosan macromolecules caused by their interactions with β-glycerophosphate. This cross-linking could restrict diffusion of chitosan macromolecules and prevent them from formation of an interpenetrating layer with mucins present on mucosal surface. According to the diffusion theory of mucoadhesion, this could reduce the mucoadhesive properties of the formulations (Khutoryanskiy, 2011).

### 3.6 Mitomycin-C *in vitro* release

**Fig. 7:**
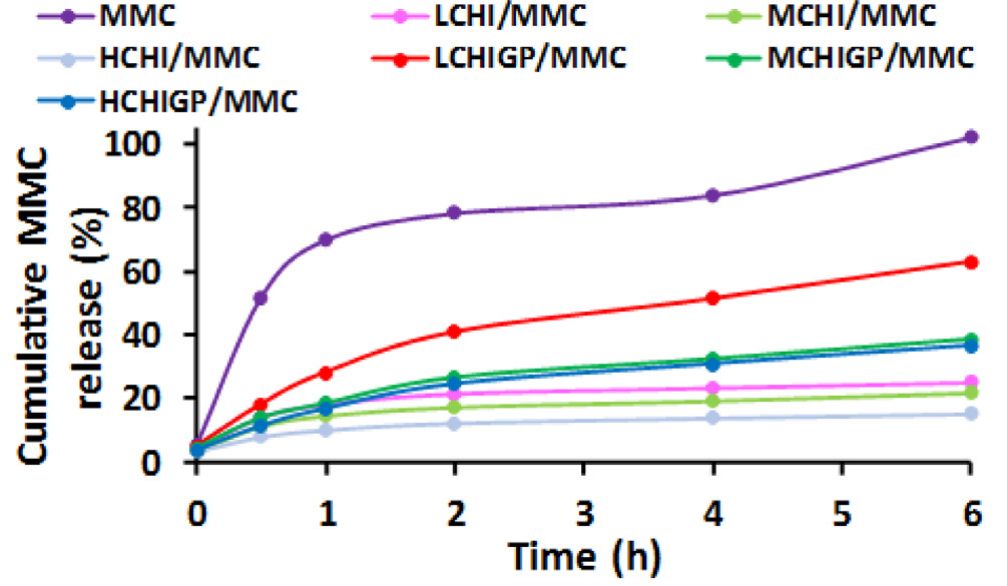
*In vitro* release profiles of mitomycin-C from free drug solution, chitosan solutions and CHIGP formulations in pH 6.2 artificial urine. Results are presented as mean values (n = 3); error bars are not shown to avoid overlapping for some samples.

Dialysis technique using semi-permeable membrane is an established method for drug release studies in intravesical drug delivery (Senyiğit et al., 2015, GuhaSarkar et al., 2017). Mitomycin-C (MMC) was chosen in the current study as a drug that is typically used intravesically to treat superficial/non-invasive bladder cancer. Fig. 7 shows the release profiles from drug solution, CHI, and CHIGP formulations.

Within the first 30 min, the release of mitomycin-C from the free drug solution, LCHI, MCHI, HCHI, LCHIGP, MCHIGP and HCHIGP samples was 52 ± 21, 14 ± 2, 11 ± 2, 8 ± 1, 18 ± 2, 14 ± 1 and 11 ± 1%, respectively. Expectedly, drug release from free mitomycin-C solution was the greatest after 30 min, which was more significant than that of CHI and CHIGP formulations (p < 0.05). After 6 h, the drug release profile for free mitomycin-C solution was 100% and it was statistically similar to that of LCHIGP (p > 0.05), inferring that *in situ* gelling systems based on low molecular weight chitosan had the least ability to sustain drug release amongst the CHIGP samples. As expected, the release from the free drug solution was faster compared to polymer-containing formulations, which is consistent with the literature on release studies involving small molecules (see Zhou et al., 2008 as an example). There was no significant difference in the drug release pattern of the pure chitosan formulations (LCHI, MCHI and HCHI), as compared at 0.5 h and 6 h (p > 0.05). This behaviour is not ideal for intravesical dosage forms, where it is desirable for a substantial amount of the therapeutic dose to be available after transurethral resection of the tumours, for superficial bladder cancer management. In contrast, there was some influence of chitosan molecular weight on the drug release from CHIGP samples. For example, a cumulative release of 63 ± 23%, 39 ± 20% and 37 ± 17% of the drug was observed over 6 h release period for LCHIGP, MCHIGP, and HCHIGP samples, respectively. This finding is in good agreement with the report by Zhou et al. (2008), where high molecular weight chitosan based gels (CHI-αβ-GP) exhibited slower adriamycin release compared to lower molecular weight polysaccharide based formulations (50% vs 70%) over 24 h. There was statistically significant difference in the cumulative amount of drug released from MCHIGP and HCHIGP compared to the free drug solution (p < 0.05). The drug release behaviour of the samples beyond 6 h was not studied because mitomycin-C degraded with prolonged exposure to artificial urine (Myers et al., 2017). The drug degradation is due to the hydrolysis of its labile ester bond in the aqueous medium. Nevertheless, drug degradation in the physiological fluid may be avoided as its release and diffusion across underlying diseased tissues will take place quickly.

LCHIGP/MMC displayed 1.6-fold and 1.7-fold increase in the amount of mitomycin-C released after 6 h release period, inferring that LCHIGP favoured an overall rapid drug release relative to MCHIGP and HCHIGP. The amount of MMC released from chitosan decorated poly-Ɛ-caprolactone nanoparticles evaluated by Bilensoy et al. (2009) was 80% in 6 h release period using phosphate buffer (pH 6.0). In a subsequent report, *in vitro* studies were carried out by the same group using a different release medium of citrate buffer but having similar pH 6.0 (Erdoğar et al., 2012), and 89%, 92% and 91% of MMC were respectively released in 15 min from chitosan, poly-l-lysine-coated and chitosan coated poly-Ɛ-caprolactone nanoparticles, inferring that the type of release medium used modulates the drug release profile rather than their pH values. Moreover, the use of physiologically relevant release medium like simulated urine used in the current study is valuable to generate reliable drug release data. These drug carriers exhibited fast drug release, which will necessitate frequent dosing that is not convenient for bladder cancer patients as their therapy is carried out in the hospital. In contrast, our MCHIGP/MMC and HCHIGP/MMC favoured sustained drug release as 39 ± 20 and 37 ± 1% of the drug were respectively released in 6 h from these formulations. This may prolong dosing interval and minimise bladder cancer recurrence. On the other hand, LCHIGP/MMC, with 63 ± 23% of drug released within 6 h, displayed a comparable profile with the best chitosan based formulations (chitosan coated PCL nanoparticles) reported by Bilensoy et al (2009).

## 4 Conclusions

*In situ* gelling systems composed of chitosan of three molecular weights and β-glycerophosphate were formulated in this work and studies for their potential applicability for intravesical delivery of mitomycin-C to treat bladder cancer were carried out. These formulations were evaluated for their ability to form gels *in situ*, rheological properties, syringeability, retention on and adhesion to the urinary bladder mucosa as well as the drug release *in vitro*.

The molecular weight of chitosan was found to modulate syringeability, gelation, mucoadhesive properties and drug release profiles of the formulations. Chitosan with the highest molecular weight (370 kDa) combined with β-glycerophosphate displayed superior resistance to urine wash-out compared to the formulations with lower molecular weights; it also provided controlled release of mitomycin-C over 6 h period.

This work showed that the mucoadhesive properties of chitosan are reduced by its formulation with β-glycerophosphate. This is related to the reduction in positive values of zeta potential for these formulations compared to chitosan alone. So, in terms of the retention of the formulations in the bladder, the use of *in situ* gelling dosage forms composed of chitosan and β-glycerophosphate did not show any superior mucoadhesive benefit over simple solutions of chitosan without gelation properties. However, the drug release pattern from chitosan solutions demonstrated that the local availability of mitomycin-C in the bladder may be limited as a maximum of 15.1–24.9% of the drug was released over the study period. Thus, HCHIGP still demonstrated superior urothelial mucoadhesive properties relative to LCHIGP and MCHIGP. Future work will explore chemical modification of chitosan prior to formulating with β-glycerophosphate to develop *in situ* gelling systems with improved mucoadhesiveness.

## Conflict of interest

The authors do not have any conflicts of interest to declare
