# Supplementary material for: Chitosan/β-glycerophosphate in situ gelling mucoadhesive systems for intravesical delivery of mitomycin-C
Source: Int J Pharm X. 2019 Feb 22;1:100007. doi: 10.1016/j.ijpx.2019.100007 (PMC6733296; doi:10.1016/j.ijpx.2019.100007)
Supplement: Supplementary data 1 [file mmc1.docx]

**Supporting information**

**Chitosan/β-glycerophosphate *in situ* gelling mucoadhesive systems for intravesical delivery of Mitomycin-C**

**Oluwadamilola M. Kolawole^a^, Wing Man Lau^b^, Vitaliy V. Khutoryanskiy^a*^**

^a^Reading School of Pharmacy, University of Reading, Reading, Berkshire, United Kingdom

^b^School of Pharmacy, The Faculty of Medical Sciences, Newcastle University, United Kingdom

*Corresponding author:

Prof. Vitaliy Khutoryanskiy, Department of Pharmacy, University of Reading, Reading, United Kingdom, RG6 6AP. Tel.: +44 (0) 118 378 6119. E-mail address: [v.khutoryanskiy@reading.ac.uk](mailto:v.khutoryanskiy@reading.ac.uk)

#
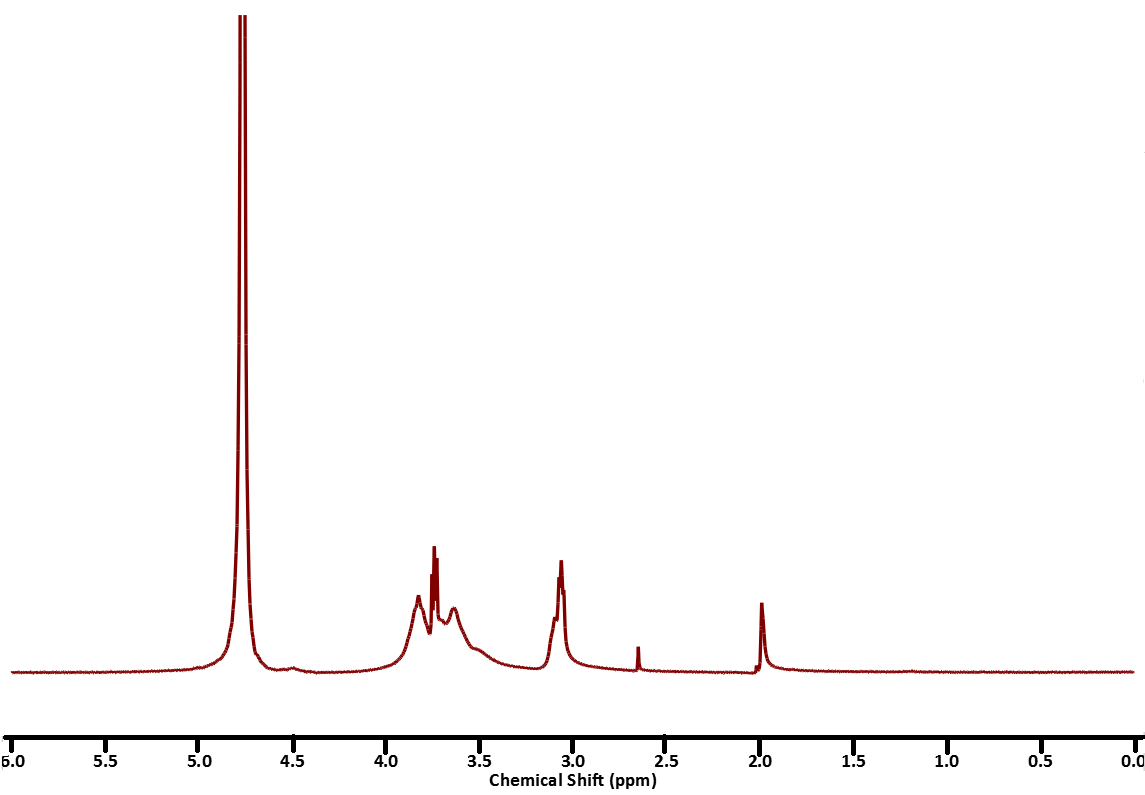

**Fig. S1.** ^1^H NMR spectra of chitosan recorded in D_2_O acidified with 1% trifluoroacetic acid. Protons for the acetylated segment of chitosan were detected at around 2 ppm while that of the deacetylated glucosamine H2-H6 protons were evident at 3.0-3.8 ppm.


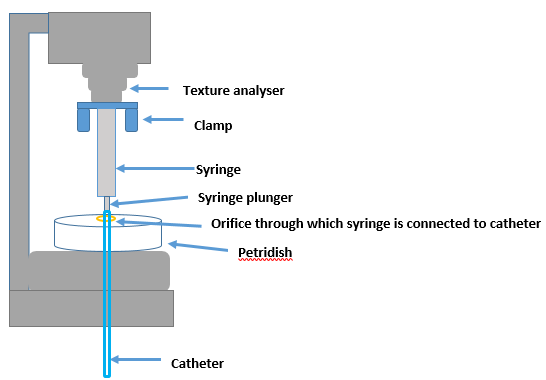


**Fig. S2.** Schematic representation of the texture analyser used to perform syringeability studies

**Fig. S3.** Schematic set up for retention studies using porcine bladder tissues and artificial urine.

**Fig. S4.** The mucosal fluorescence retention profile of FITC-dextran, chitosan and chitosan/β-glycerophosphate systems on porcine bladder tissues evaluated using ImageJ software and WO_50_ values calculated based on the polynomial fit of the graphs_._ Result presented as mean ± standard deviation, n = 3, error bars not shown as they will overlap.

#

**Fig. S5.** Adhesion of CHI (1% w/v) and CHIGP hydrogels to porcine bladder: Schematic representation of texture analyser demonstrating bioadhesion evaluation.

# **Fig.S6.** Exemplar pattern of the detachment of CHIGP hydrogel from porcine ladder mucosa during the mucoadhesion studies carried out using the Texture Analyser (Stable Micro Systems Ltd, UK)

**Fig. S7.** Exemplar HPLC-UV chromatogram of mitomycin-C loaded chitosan and chitosan/β-glycerophosphate systems, with mitomycin-C eluting at about 10 min and salt constituents of the artificial urine eluting at 0.9; 1.2 and 4 min.


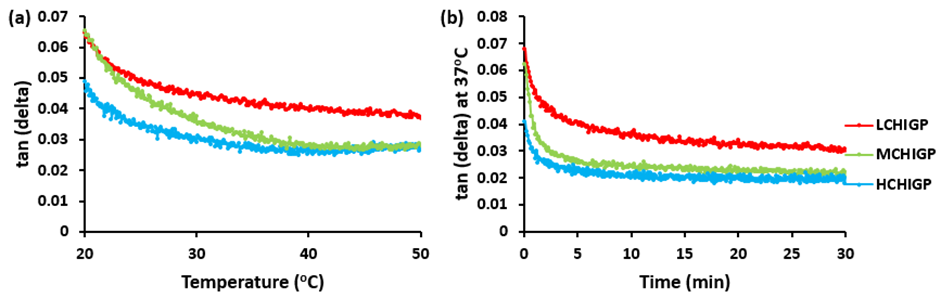


**Fig. S8.** Tanδ rheological profiles for temperature-dependent (a) and time-dependent changes (b) of LCHIGP (red), MCHIGP (green) and HCHIGP (blue)
